# Tissue Interleukin-33: A Novel Potential Regulator of Innate Immunity and Biomarker of Disease Severity in Chronic Rhinosinusitis with Nasal Polyps

**DOI:** 10.3390/jcm12247537

**Published:** 2023-12-06

**Authors:** Ioana Maria Porfire (Irimia), Ioana Berindan-Neagoe, Livia Budisan, Daniel-Corneliu Leucuta, Anda Gata, Aurelian Costin Minoiu, Bogdan Alexandru Georgescu, Bogdan Florin Covaliu, Silviu Albu

**Affiliations:** 1 IInd Department of Otorhinolaryngology, ‘Iuliu Hatieganu’ University of Medicine and Pharmacy, 400349 Cluj-Napoca, Romania; silviualbu63@gmail.com; 2 Research Center for Functional Genomics, Biomedicine and Translational Medicine, “Iuliu Hatieganu” University of Medicine and Pharmacy, 400349 Cluj-Napoca, Romania; ioananeagoe29@gmail.com (I.B.-N.); liviuta.petrisor@umfcluj.ro (L.B.); 3 Medical Informatics and Biostatistics Department, University of Medicine and Pharmacy “Iuliu Hatieganu”, 400349 Cluj-Napoca, Romania; dleucuta@umfcluj.ro; 4 Diagnostical and Interventional Radiology Department, Carol Davila University of Medicine and Pharmacy, 020021 Bucharest, Romania; costin.minoiu@gmail.com; 5 Faculty of Medicine, ‘Ovidius’ University, 900527 Constanta, Romania; orl.dr.alexgeorgescu@gmail.com; 6 IVth Department of Community Medicine, ‘Iuliu Hatieganu’ University of Medicine and Pharmacy, 400349 Cluj-Napoca, Romania; bogdancovaliu@gmail.com

**Keywords:** CRSwNP, nasal polyp, IL-33, osteitis, eosinophilia, Lund-Mackay score, nasal endoscopy

## Abstract

Background: Chronic rhinosinusitis with nasal polyps (CRSwNP) is a disease of real interest for researchers due to its heterogenicity and complex pathophysiological mechanisms. Identification of the factors that ensure success after treatment represents one of the main challenges in CRSwNP research. No consensus in this direction has been reached so far. Biomarkers for poor outcomes have been noted, but nonetheless, their prognostic value has not been extensively investigated, and needs to be sought. We aimed to evaluate the correlation between potential prognostic predictors for recalcitrant disease in patients with CRSwNP. Methods: The study group consisted of CRSwNP patients who underwent surgical treatment and nasal polyp (NP) tissue sampling. The preoperative workup included Lund–Mackay assessment, nasal endoscopy, eosinophil blood count, asthma, and environmental allergy questionnaire. Postoperatively, in subjects with poor outcomes, imagistic osteitis severity was evaluated, and IL-33 expression was measured. Results: IL-33 expression in NP was positively and significantly correlated with postoperative osteitis on CT scans (*p* = 0.01). Furthermore, high osteitis CT scores were related to high blood eosinophilia (*p* = 0.01). A positive strong correlation was found between postoperative osteitis and the Lund–Mackay preoperative score (*p* = 0.01), as well as the nasal endoscopy score (*p* = 0.01). Conclusions: Our research analyzed the levels of polyp IL-33, relative to blood eosinophilia, overall disease severity score, and osteitis severity, in patients with CRSwNP. These variables are prognostic predictors for poor outcomes and recalcitrant disease. Considering the importance of bone involvement in CRSwNP, this research aims to provide a better insight into the correlations of osteitis with clinical and biological factors.

## 1. Introduction

### 1.1. Inflammatory Cytokines; Overview of Th2 Inflammatory Pathways in the Pathogenesis of Nasal Polyps

Chronic rhinosinusitis is a heterogeneous inflammatory disease divided into two phenotypes: chronic rhinosinusitis with nasal polyp (CRSwNP) and chronic rhinosinusitis without nasal polyp (CRSsNP). CRSwNP has three endotypes: One is the Th2 endotype, which is characterized by the increased production of Th2 cytokines associated with type-2 immune response, and association with eosinophilic infiltration. Additionally, the T1 and T3 endotypes are associated with neutrophilic infiltration [1].

Despite advances in medical and surgical therapy, CRSwNP remains challenging to treat. ICAR-RS-2021 [2] strives to improve the understanding of the disease and medical or surgical treatment of CRSwNP through evidence-based recommendations. A significant rate of recurrence is present, even though the disease benefits from endoscopic sinus surgery and long-term usage of corticosteroids. Up to 50% of polypoid recurrences and 30% of the courses of revision surgery eventuate the persistent inflammatory state, so better understanding and development of new therapeutical approaches are mainstays for the researchers [3].

Specific factors that trigger an inflammatory response in the pathogenesis of nasal polyps have been identified. The endotype of CRSwNP presents mainly with Th2-induced inflammation, and it has been associated with refractoriness, comorbidities, and resistance to steroid therapy. Epithelial-derived innate cytokines, including IL-33, promote Th2 responses via the development of innate lymphoid cells and play a crucial role in the development of CRSwNP by inducing various pathways. Innate immunity and epithelial-derived innate cytokines need to be investigated more as Th2-targeted biomarkers in the effort to unravel the mechanisms of refractory CRSwNP [4].

### 1.2. Assessment of CRSwNP-Associated Osteitis; Radiological Features in Computed Tomography

Several animal studies, including our previous research, have demonstrated a link between experimentally induced rhinosinusitis and chronic inflammation of the bony middle turbinate and the ethmoid and maxillary sinus bony walls [5,6].

The term osteitis has been used to describe the bony thickening of sinus walls present in chronic rhinosinusitis (CRS). Since the first detection of bone involvement in early studies, contemporary studies have recognized osteitis as a common factor in recalcitrant CRS, one which is associated with increased disease severity and an increased number of surgeries. Osteitis is an outcome of neo-osteogenesis, rather than inflammatory processes, in CRS patients without a prior history of surgery [7]. There is increased recognition of the high prevalence of osteitis changes affecting the bony framework of the sinuses in patients with chronic rhinosinusitis, with or without nasal polyps. However, their grading, clinical significance, and management remain controversial. Recent findings demonstrate that osteitis is more often present in patients with extended radiological disease and in patients undergoing revision surgery. The number of previous surgeries appears to be closely correlated with the extent of sinusitis, although it is not clear whether that is a direct or a secondary association [5]. Histological samples of patients with rhinosinusitis were also analyzed by Lee et al. [8], who found pathological evidence of osteitis in 53% of their samples, ranging from 6.7% in those undergoing primary surgery to 58% in patients having revision endoscopic sinus surgery (ESS). At the same time, CT evaluation showed a prevalence of bony involvement in CRS of around 51%, with a higher prevalence in patients with previous sinus surgery (76%) than in patients with primary surgery (36%) [7].

A computed tomography (CT) scan of the paranasal sinuses is the gold standard diagnostic radiological tool for chronic rhinosinusitis (CRS), used to evaluate the disease’s extent and its complications. In most patients, current clinical practice recommends a CT scan in addition to the diagnostic modality of choice to evaluate the presence and the extent of osteitis in patients with CRS. Hence, osteitis is almost always a radiological diagnosis, but is occasionally confirmed with pathological samples [5,9].

### 1.3. Correlation between Radiological Severity and Outcomes of Lund–Mackay Scores, Eosinophilia, and Nasal Endoscopy in CRSwNP

Several studies have confirmed a clear correlation between radiological severity and the extent of CRS, as measured with the Lund–Mackay grading system, for osteitis. Lund–Mackay scores and endoscopic scores have been shown to correlate with osteitis [6]. There is evidence for the association of osteitis with revision surgeries and CRS severity in terms of higher Lund–Mackay scores [7]. Moreover, previous ESS was associated with high osteitis prevalence, greater endoscopic score severity, worse computed tomography grading, and poor outcomes (Lee et al. 2006) [8]. Also, there is evidence of osteitis being typically associated with nasal polyps, eosinophilia, and recurrent CRS; however, its roles and pathogenesis remain unclear. (Lee et al. 2006 [8]; Khalmuratrova et al. 2020 [10]).

### 1.4. Relevance of the Study

Pathogenesis of nasal polyps has been extensively studied; epithelial derived innate cytokines, including IL-33, promote Th2 responses via the development of innate lymphoid cells to ensure the persistence of disease, even when the mucosa is either treated medically or removed [5]. We aimed to study tissue IL-33 in CRSwNP, using an enzyme-linked immunosorbent assay (ELISA) to investigate its involvement as a novel potential regulator of innate immunity, and biomarker of disease severity, in chronic rhinosinusitis with nasal polyps. Notably, while there is evidence of increased serum IL-33 levels in CRS, there is very little research on tissue IL-33. Moreover, references regarding the evaluation of inflammation, both at the immunological and imaging level, as well as the association of IL-33 with the severity of osteitis, are extremely sparse. Recent studies have associated osteitis with nasal polyps and tissue eosinophilia and the Lund–Mackay score. While CT has become a staple in osteitis assessment, the standards for grading osteitis severity remain at an experimental stage.

## 2. Materials and Methods

### 2.1. Study Design and Protocol

Our research was carried out with a cohort of 86 Caucasian patients (57 men, 29 women) aged between 18 and 80 years old, and diagnosed with CRSwNP according to the diagnostic criteria of the *European Position Paper on Rhinosinusitis and Nasal Polyps* (EPOS) [11]. The decision to undergo surgery, as well as all clinical investigations, were made following the course of CRSwNP disease management, and they were not part of this study’s protocol. Out of the 86 subjects, only patients with CRSwNP with poor outcomes after maximal medical treatment, according to nasal endoscopies and Lund–Mackay preoperative assessment, were proposed for primary surgery and were included; *n* = 44 (21 men, 13 women). All patients with revision surgery were excluded. Also, use of systemic corticosteroids or macrolide treatment in the previous month, concurrent immunomodulatory therapy, previous nasal trauma, and sinonasal malignancies were considered to be exclusion criteria. The preoperative workup included nasal endoscopy; CT scan with Lund–Mackay score for disease extension; blood eosinophils count; and questionnaires for comorbid conditions such as asthma, environmental allergies, and other pathologies, as well as demographic data. Endoscopic sinus surgery (ESS) was carried out as a choice treatment therapy, and a nasal polyp sample was harvested. Tissue IL-33 expression was quantified, and imaging native cranial CT scans were performed at 18 months post-operation. Correlations between the tissue IL-33 levels, osteitis severity score, blood eosinophilia, and Lund–Mackay overall disease severity score were subsequently evaluated.

The study was conducted in accordance with the guidelines of the Declaration of Helsinki. All participants provided signed written informed consent to their participation in the study. All patients agreed to the collection of blood and mucosal samples for histopathology and IL-33 quantitative analysis, as well as pre- and postoperative evaluation. Access decisions regarding participants’ personal data respected patient confidentiality and privacy. The harvesting and measurement protocols for this study were approved by the Iuliu Hatieganu University of Medicine and Pharmacy Ethics Committee under approvals No. 590/10.12.2019 and No. 146/06.06.2022.

### 2.2. Patient Group Selection

The patients were recruited from the Department of Otorhinolaryngology of the CF Cluj Clinical Hospital, Romania, between March 2020 and July 2021.

The research design, as well as the inclusion and exclusion criteria, are presented herein in Table 1. In order to maintain the homogeneity of the study population, we decided to exclude all factors which could influence IL-33 levels or the degree of osteitis.

Additionally, data as to demographics (i.e., age and gender) and comorbid conditions such as asthma and environmental allergies were collected. High blood eosinophilia was defined as having values of over 5% of total white blood cells. Data from the quality-of-life SNOT-22 questionnaire, endoscopy scores, and polyp sizes rated using the Lildholdt scale [12] were recorded (Table 2).

### 2.3. Sampling

Preoperative patient preparation with anesthesia evaluation was performed. After induction of general anesthesia by oro-tracheal intubation, the nasal vestibule was cleaned with iodine. Subsequently, under visual control using 0-degree endoscope, a local vasoconstricting agent was applied within the mucosa of the nasal cavities. After carefully exploring the nasal corridor and middle meatus, the polypoid tissue was visualized. The polyps were removed using polyp forceps and through-cutting instruments. Two mucosal samples were obtained from the nasal polyp from the middle meatus of each patient; the samples for histopathology were stored in 7% formaldehyde. The samples for IL-33 quantitative analysis were stored, immediately after collection, at −80 °C until analysis. For all 44 samples the diagnosis of CRSwNP was confirmed by the histopathology department of the hospital.

### 2.4. Assessing Tissue IL-33 in Nasal Polyp Specimens

#### 2.4.1. Extraction of Total Proteins

For protein extraction, we used 44 fresh frozen samples severally retrieved from each individual with nasal polyposis. The tissue samples were rinsed with cold phosphate buffer solution (PBS) and homogenized in 1 mL phosphate buffer solution PBS 1X (0.01 mol/L, pH = 7–7.2). Then, 600 µL PBS 1X were added and the samples were frozen in liquid nitrogen. Aliquots of tissue homogenates were incubated 30 min at 4 °C on an ice shaker and centrifugated at 12,000 rpm for 10 min at 40 °C. Protein supernatants were taken out, transferred, and preserved in new polypropylene tubes kept at −20 °C.

#### 2.4.2. Enzyme-Linked Immunoassay (ELISA) Protein Quantification

Protein quantification was measured using a spectrophotometric method with a NanoDrop 2000c (Thermo Fischer, Waltham, MA, USA). The protein samples were diluted with PBS 1X for a final concentration of 100 µg/100 µL. Further, human IL-33 determination was performed by the ELISA method using a Human Interleukin 33 ELISA kit (Abbexa (Cambridge, UK), catalog no: abx152042) according to the recommended protocol. Separately, IL-33 quantification was performed using GraphPad Prism 6 (GraphPad, Boston, MA, USA).

The Human Interleukin 33 (IL33) ELISA Kit is an ELISA Kit for the in vitro quantitative measurement of human interleukin 33 (IL33) concentrations in serum, plasma, tissue homogenates, cell lysates, cell culture supernatants, and other biological fluids. This assay has a high level of sensitivity and excellent specificity for the detection of interleukin 33 (IL33). No significant cross-reactivity or interference between interleukin 33 (IL33) and its analogues was observed.

### 2.5. CT Scan and Osteitis Score Assessments

#### 2.5.1. Osteitis in Patients with CRSwNP

It was demonstrated that the overlying sinonasal mucosa plays a vital role in the inflammatory process; moreover, it was found that, through the Haversian system, the inflammatory mediators may spread, even to non-adjacent bony structures. Thus, osteitis, which is recognized as a common factor in recalcitrant chronic rhinosinusitis, may be the result of both mucosal and bony involvement, which may contribute to the onset, dissemination, and persistence of the inflammatory status in chronic rhinosinusitis (CRS) [7,13,14].

#### 2.5.2. Computed Tomography (CT) Scan in Clinical Practice in CRSwNP

In most studies, and certainly in current clinical practice, a CT scan is the modality of choice to evaluate the presence and extent of osteitis in patients with CRS. Hence, osteitis is, almost always, a radiological diagnosis, occasionally confirmed with pathological specimens taken during surgery.

Over time, several osteitis scoring systems have been developed, such as the Kennedy osteitis score, the modified global osteitis score, CT thickness grading of osteitis, and the rating of bone density in Hounsfield Units (HU) [7,8,15,16,17]. In our research, we applied the scale developed by Georgalas et al., due to the complexity of its structure, its simplicity of evaluation, and its direct correlation with the Lund–Mackay score. The osteitis was thus classified as not significant (<5), mild (5–20), moderate (20–35), or severe (higher than 35), as presented in Table 3 [5,18].

**Figure 1:**
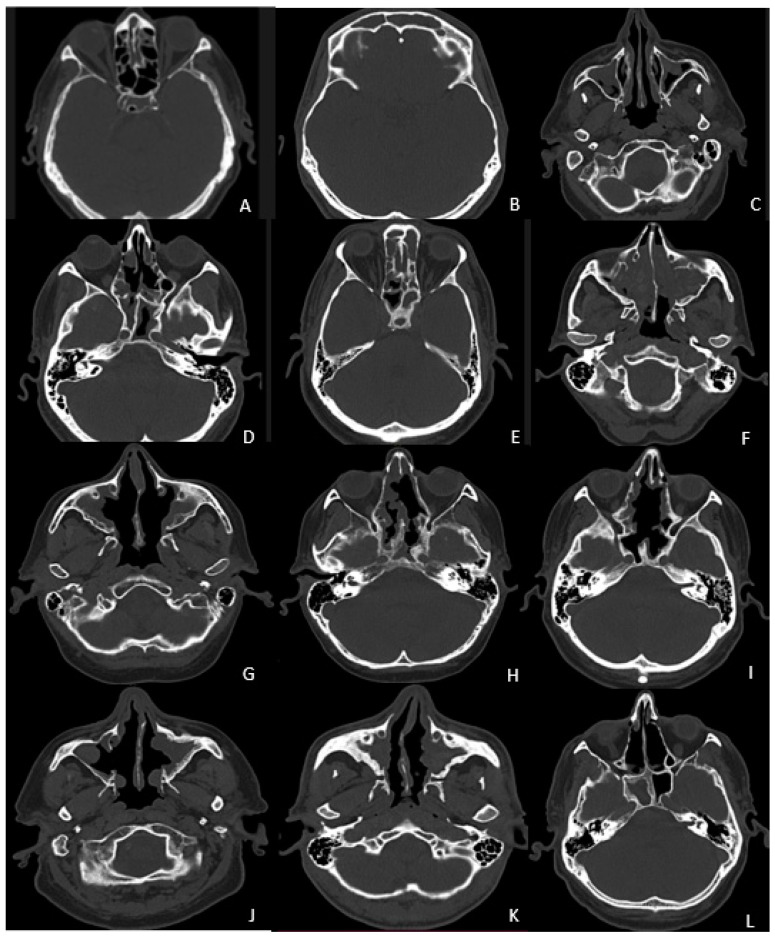
Evidence of grade 1 osteitis of right anterior ethmoid and sphenoid sinuses (**A**,**D**), and left frontal (**B**) and maxillary sinuses (**C**) in a patient who has undergone previous sinus procedure for chronic rhinosinusitis with nasal polyps, at 12-month postoperative evaluation. Grade 2 osteitis calculated according to the same severity scale, in different patients (posterior right ethmoidal sinus (**E**), and also in both maxillary sinuses (**F**)). Evidence of grade 3 osteitis in both maxillary and sphenoidal sinuses (**G**,**H**). Features of grade 4 left sphenoid and maxillary sinuses (**I**,**J**) and grade 5 osteitis within the right maxillary and right sphenoid sinuses (**K**,**L**).

Several studies have confirmed a clear correlation between the radiological severity and extent of CRS, as measured with the Lund–Mackay grading system, and osteitis. Lund–Mackay scores, as well as endoscopic scores, have been shown to be correlated with osteitis [6]. The global osteitis grading scale (GOSS) is a novel validated composite grading system that measures the extent and severity of osteitis and assesses the area of maximal thickness of the bony wall for each sinus. According to Georgalas et al. [5], the grading using this scale is easy to perform (usually 2–3 min per patient) and gives reproducible results (Figure 1).

### 2.6. Statistical Analysis

To assess the correlations between different quantitative variables, we used a Spearman correlation coefficient and 95% bootstrapped confidence intervals. A multiple linear regression model was fitted to predict the CT osteitis score, having as a variable of interest IL-33. The model was adjusted using confounding variables for the presence of asthma and the endoscopy sum score. The adjustment variables were chosen, before fitting the model, based on their likelihood to be associated with the CT osteitis score from the clinical rationale. To prevent overfitting, we chose only three independent variables in the model to observe a limit of 10 subjects per variable (the degrees of liberty of the model being 40). The normality of the residuals, the presence of heteroskedasticity, the presence of multicollinearity, and the linearity of quantitative independent variables with the dependent variable adjusted for the presence of the confounders were all checked as assumptions of the model. The model coefficients, their 95% confidence intervals, and *p*-values were reported. For all statistical analyses the R environment for statistical computing and graphics (R Foundation for Statistical Computing, Vienna, Austria), version 4.1.3 [R Core Team. R: A Language and Environment for Statistical Computing. R Foundation for Statistical Computing: Vienna, Austria; 2023.] was used. A 0.05 limit for statistical significance and two-tailed *p*-values were used for all statistical analyses.

## 3. Results

### 3.1. Correlation between CT Osteitis Score and IL-33 Expression (pg/mL)

**Figure 2:**
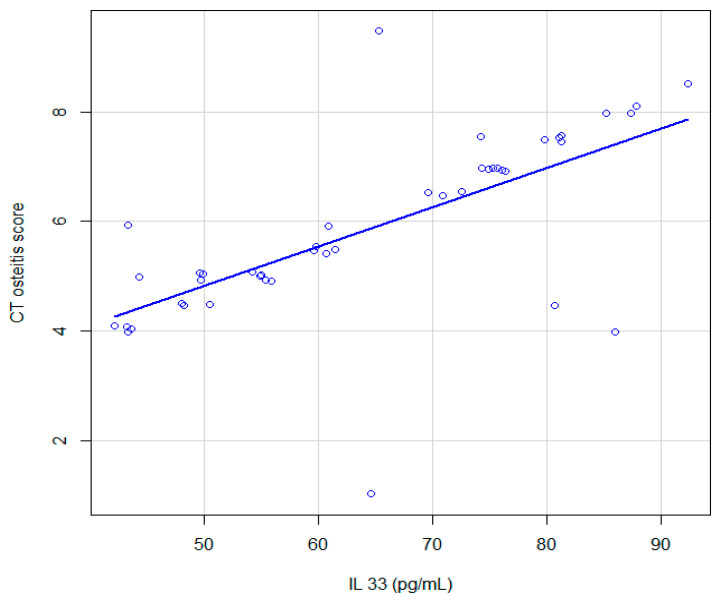
Graphic representation of the association between CT osteitis score and expression of IL-33 pg/mL in patients with CRSwNP; continuous line, locally estimated scatterplot smoothing, with 95% confidence interval.

A Spearman correlation analysis was run to determine the relationship between the computed tomography osteitis score and interleukin-33 levels as depicted in Figure 2. There was a strong, positive correlation between the computed tomography osteitis score and interleukin-33 levels, which was statistically significant, rs(42) = 0.73, 95% CI [0.44, 0.92], *p* < 0.001. The median for IL-33 is 60.24 (interquartile range: 49.5–72.97), and the range 29.42–107.88.

### 3.2. Correlation between CT Osteitis Score and Eosinophiles (10^9^ L)

**Figure 3:**
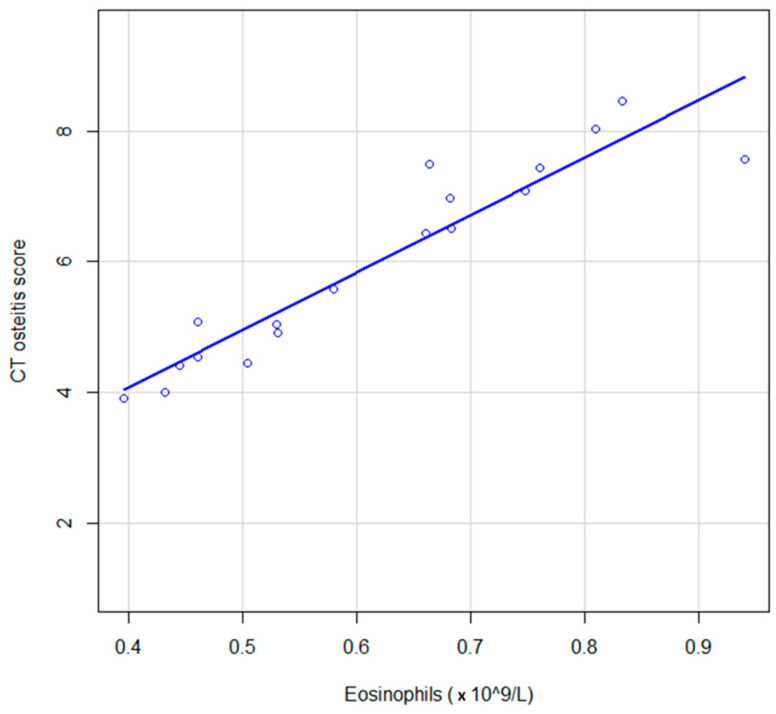
Graphic representation of the association between CT osteitis score and blood eosinophiles (10^9^ L) in patients with CRSwNP; continuous line, locally estimated scatterplot smoothing, with 95% confidence interval.

The correlation between CT osteitis score and eosinophiles (10^9^ L) was evaluated, and a Spearman correlation coefficient = 0.95 (95% CI 0.88–0.99) was obtained, associated with the significance test *p* ≤ 0.001 (Figure 3). The correlation found is very good.

### 3.3. Correlation between CT Osteitis Score and Lund–Mackay Score

**Figure 4:**
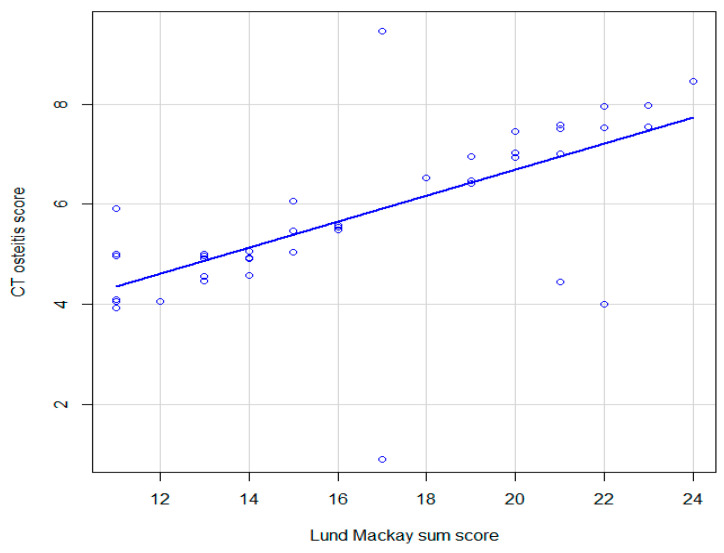
Graphic representation of the association between CT osteitis score and Lund–Mackay score in patients with CRSwNP; continuous line, locally estimated scatterplot smoothing, with 95% confidence interval.

A Spearman correlation analysis was conducted to assess the strength of the relationship between the computed tomography (CT) osteitis score and the Lund–Mackay sum score. The results of the correlational analysis revealed a significant positive correlation between the CT osteitis Score and the Lund–Mackay sum score, with a Spearman’s rho value of 0.72 (95% confidence interval: 0.45–0.9), *p* < 0.001 (Figure 4). This suggests that as the CT osteitis score increases, there is a corresponding increase in the Lund–Mackay sum score, indicating a good strength of association between the two variables.

### 3.4. Correlation between Nasal Endoscopy Score and IL-33 Expression pg/mL

**Figure 5:**
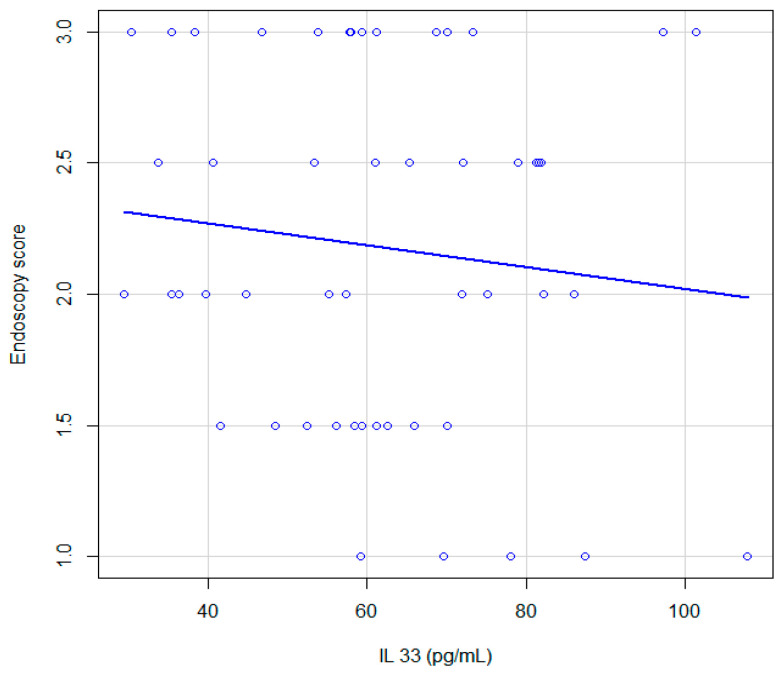
Graphic representation of the association between endoscopy score and IL-33 pg/mL.

The correlation between endoscopy score and IL-33 pg/mL expression was evaluated and a Spearman correlation coefficient = 0.6 (95% CI 0.35–0.76) was obtained, associated with the significance test *p* ≤ 0.001 (Figure 5). The correlation found is good.

### 3.5. Multiple Linear Regression Model Testing CT Osteitis Score, IL-33, Asthma and Endoscopy Score

After performing the univariate analyses regarding relations between CT osteitis score and other predictors, we built a multiple linear regression model. The dependent variable was the CT osteitis score, and the variable of interest was IL-33, adjusted for the presence of asthma and endoscopy (Table 4. We observed that higher values of IL-33 are associated with higher values of the CT osteitis score, the relation being statistically significant (B = 0.086, t (40) = 5.505, *p* < 0.001, 95% CI [0.054, 0.117]). Asthma and endoscopy scores were not statistically significantly associated with the dependent variable (B = −0.672, t (40) = −1.623, *p* = 0.112, 95% CI [−1.509, 0.165]); similarly, the endoscopy score was not found to be a significant predictor (B = −0.160, t (40) = −0.977, *p* = 0.334, 95% CI [−0.490, 0.171]). The multiple linear regression model was statistically significant (*p* < 0.001), with an adjusted determination coefficient of 0.45. The constant for the model was B = 1.223 with a standard error of 0.830, which was not statistically significant (t (40) = 1.473, *p* = 0.149, 95% CI [−0.455, 2.902]).

## 4. Discussion

An important challenge in researching CRS is the identification of the factors that ensure success after treatment. No consensus in this direction has been reached so far. Both EPOS (the recent *European Position Statement on Rhinosinusitis*) and the *International Consensus* statement recommend consideration of patient-reported outcome measures (PROMs), endoscopic, and/or radiographic variables. Despite the attention given over the past decade to the recurrences after maximal medical and surgical treatment, multiple knowledge gaps remain, particularly within the realms of developing better diagnostic procedures by endotype validation and targeted therapeutic strategies, and discovering biomarkers that directly respond to targeted therapeutics and that may predict efficacy, advancing the personalized treatment of CRS.

The 2021 *International Consensus Statement on Allergy and Rhinology in Rhinosinusitis* and the 2020 *European Position Paper on Chronic Rhinosinusitis and Nasal Polyps* developed guidance protocols for optimal disease management: intranasal corticosteroids, antibiotics, and endoscopic sinus surgery. Additionally, recurrence-contributing factors such as anatomic factors, neo-osteogenesis, innate immunity, allergies, immunologic defects, immunodeficiencies, biofilms, fungus, gastroesophageal reflux, superantigens, microbiome disturbance, occupational and environmental factors, viruses, or vitamin-D deficiency are largely debated without a resulting clear conclusion in terms of the extent to which they are implicated in the relapse process [2,11,17].

Prior studies on CRSwNP disease severity describe individual clinical variables, demographics (age and sex), radiological features, nasal endoscopy evaluation, asthma status, eosinophil density measures, and specific cytokines as prognostic predictors or biomarkers for poor outcomes and recalcitrant disease. Nonetheless, their prognostic value has yet to be extensively investigated and needs to be determined [17]. These variables are prognostic predictors for poor outcomes and recalcitrant disease. Considering the importance of the direct correlations our research has demonstrated between the interleukin (IL)-33 overexpression in NP, hypereosinophilia, overall disease severity score, and bone involvement in CRSwNP, this research aims to provide better insight into the correlations of osteitis with clinical and biological factors.

Over time, numerous cytokines were studied as biomarkers associated with CRSwNP (IL-4, IL-5, IL-6, IL-10, IL-21, interferon-gamma (IF γ), and tumor necrosis factor alpha (TNFα)) [19,20,21]. IL-33 is an IL-1 family cytokine. It is a newly discovered cytokine, considered important not only through the activation of inflammation but during tissue injury associated with necrosis. IL-33 is a tissue-derived nuclear cytokine, abundantly expressed in epithelial cells during homeostasis and inflammation, and now considered to be crucial for the development of allergic diseases, chronic allergies, fibrosis, infections, and inflammatory disorders, and specifically in the pathogenesis of CRSwNP [18,19,20,22].

It becomes involved in host defense by inducing Th2 cytokine production and binding specifically to its receptor suppression of tumorigenicity 2 (ST2), forming the IL-33/ ST2 axis [18,19]. In particular, IL-33 is a key cytokine for inducing and activating ILCs (innate lymphoid cells). Genetic polymorphism in ST2 or IL-33 was found in patients with rhinitis and rhinosinusitis.

In particular, IL-33 is a key cytokine for inducing and activating ILCs. Innate immunity and epithelial-derived innate cytokines need to be investigated more as Th2-targeted biomarkers in efforts to unravel the mechanism of refractory CRSwNP. Serum IL-33 levels were significantly higher in patients with rhinitis compared to healthy subjects. Notably, the number of IL-33-responding ILCs was also increased in the nasal polyps of patients with rhinosinusitis [18]. Epithelial-derived innate cytokines, including IL-33, have the function of an alarmin and promote Th2 responses via the development of group-2 innate lymphoid cells (ILC2s) [4,20]. ST2 was over-expressed in ILCs from CRSwNP tissues compared with chronic rhinosinusitis without nasal polyp (CRSsNP), and ST2-positive ILCs were a source of IL-13 in response to IL-33 [4]. Shaw et al. [23] found no significant difference in the relative expression of IL-33 in the inflamed sinus mucosa from patients with CRSwNP. Contrastingly, a high level of expression of IL-33 was present in tissue from patients with CRSwNP, CRSsNP and healthy subjects, and the authors have raised the possibility that IL-33 could interact with an ST2 cell population within the polyp-inflamed mucosa. Even though Ryu et al. [4] demonstrated the ST2 cell population was over-expressed in CRSwNP tissues compared with chronic rhinosinusitis without nasal polyp (CRSsNP), and ST2-positive ILCs were a source of IL-13 in response to IL-33, there are still controversies about the expression levels of IL-33 in nasal polyp tissues. This may be associated with the cleaved IL-33 molecules strongly showing more biological activity rather than full-length IL-33 concentration. Therefore, we need to know information about the levels of IL-33 in human samples associated with different diseases to apply IL-33 to a clinical setting as a biomarker [4]. Furthermore, targeting these key cytokines responsible for Th2 immunological responses may offer practical strategies for reducing Th2 inflammation and disease burden [24,25].

Additionally, a recent study by Blizniewska et al. [26] evaluated IL-33 and corroborated the result with computed tomography according to Lund–Mackay scores. The study concluded that IL-33 levels may be an essential marker in the classification of CRSwNP, consistent with disease severity. In addition, IL-33 levels may be helpful in predicting CRSwNP severity. Other selected inflammatory cytokines may contribute to early recognition of the disease. Several studies have confirmed a clear correlation between radiological severity and extent of CRS, as measured with Lund–Mackay grading system, and osteitis. As a noninvasive parameter, the Lund–Mackay scores on CT scans, as well as endoscopic scores, have been shown to be correlated with osteitis [6] and considered to be predictors of eosinophilic status in CRSwNP [27]. Our research was in line with the recommendations on CRSwNP disease outcomes from both the *European Position Statement on Rhinosinusitis* and the *International Consensus* statement, so we took into consideration patient-reported outcome measures (PROMs) such as generic health-related quality-of-life scores and endoscopic and radiographic variables [2,11]. In our study population, elevated expressions of IL-33 levels within the inflamed nasal mucosa were directly correlated with computed tomography osteitis scores, and their mean values were statistically significant. Moreover, our results suggest that as the CT osteitis score increases, there is a corresponding increase in the Lund–Mackay preoperative score, indicating a good strength of association between the two variables.

CRSwNP patients, particularly those resistant to treatment, were associated with increased levels of tissue and blood eosinophilia and concomitant IL-33 upregulation in nasal polyp tissues [28]. Mucosal eosinophilic status is in direct correlation with the severity and outcome of CRSwNP; also, peripheral eosinophilia is strongly correlated with the mucosal eosinophil count, so blood eosinophilia may be considered an alternative for CRS evaluation [29,30,31]. Moreover, according to Tokunaga et al. [32], blood eosinophilia and the extent of disease on computed tomography scan scores correlate with the rate of recurrence and refractoriness. IL-33 upregulation was correlated with increased eosinophil counts and endoscopy scores and plays a vital role in CRSwNP through its effects in mediating eosinophilic infiltration [33]. In our study cohort, eosinophilia was diagnosed based on peripheral blood eosinophils values. Our results were significantly correlated with high Lund–Mackay scores, CT scan osteitis severity, and endoscopic scores; however, they did not reach statistical significance for asthma status. Also, we demonstrated that as eosinophil levels increase, the CT osteitis score tends to increase. Given the strength of the correlation coefficient, the association between these two variables can be described as very strong.

Furthermore, in terms of predictability, we showed that IL-33 was a significant predictor of osteitis severity, but we did not observe the asthma status and endoscopic scores to be significant predictors. Our findings are consistent with the results in the recently published literature. Notably, most of the research evaluates serum IL-33 levels versus tissue IL-33, so part of the difference in terms of the consistency of the results with the literature can be attributed to this fact. For example, Zhang et al. [34] suggested that serum IL-33 levels were upregulated in CRSwNP patients and related to the degree of mucosal eosinophil infiltration and postoperative recurrence. Thus, IL-33 might serve as an objective biomarker for predicting recurrence in CRSwNP. On the other hand, for instance, some researchers even suggest that IL-33 is present in NP tissue but is not upregulated [35,36]. Song et al. [37] correlate IL-33 expression with eosinophil count and endoscopy score, while there is no correlation with Lund–Mackay scores. Ozturan et al. [38] even demonstrate a negative correlation between serum IL-33 and Lund– Mackay scores.In CRS associated with Th2 response, recent recommendations, in conjunction with continuous improvement of tissue testing methods, consider it advisable to search for new biomarkers whose levels can be increased. Even though they may not be pathognomonic for CRS, elevated blood eosinophilia and IL-33, along with other cytokines’ overexpression, may allow the assessment of tissue remodeling in the chronic processes of paranasal sinuses [20]. Additionally, recent clinical approaches have shown the favorable effects of monoclonal antibodies against IL-33 in eosinophilic nasal polyps. This suggests that they may be novel therapeutic strategies for CRSwNP [24]. Nonetheless, anti-IL-33 may provide a new treatment strategy for targeting infiltrating neutrophils in CRSwNP due to the demonstrated role that IL-33 has in neutrophil recruitment.

### Relevance and Limitations of the Research

Our results are in line with other expression studies existing within the literature. Although some studies evaluate IL-33 in serum from CRSwNP subjects, there are few studies regarding IL-33 in polyp tissue, and the results are inconsistent. Notably, the lack of experience in IL-33 determinations from human tissue may be a limitation in the interpretation of results.

For all patients included in the study cohort, a histopathologic CRSwNP diagnosis was obtained; this was the reason we did not discuss the eventual false positive results in IL-33 expression. However, in patients with associated asthma (*n* = 13, 29.54%) and allergies (*n* = 16%) who may be subjected to false positive IL-33 overexpression, we cannot demonstrate to what extent the high values of IL-33 are associated with CRS or allergies, or, respectively, asthma.

Our research included only patients with CRSwNP who underwent primary surgical treatment, and who demonstrated a tendency to be recalcitrant based on postoperative clinical evaluation through nasal endoscopy. Therefore, the results cannot be extrapolated to other severity groups. Yet, our results may serve as a starting point for other studies.

## 5. Conclusions

The present study is one of the few that simultaneously analyze and correlate the levels of IL-33 from NP tissue in CRSwNP human subjects with blood eosinophilia and overall disease severity score. Ongoing studies on the role played by IL-33 are accumulating and suggest the possibility of novel therapeutic strategies for treating CRSwNP. On the other hand, considering the increasing importance of bone involvement in CRSwNP, this research aims to provide better insight into the correlation between osteitis and biological factors. Moreover, evaluating the Lund–Mackay preoperative scoring system, along with nasal endoscopy score and GOSS for osteitis severity, the possibility that GOSS may be a suitable and convenient scoring system for clinical practice in grading osteitis was raised. Due to the results of our research, we have contributed to the scarce number of conducted studies on these issues. Further studies will better clarify the role of IL-33 as a novel potential regulator of innate immunity and a biomarker of disease severity in chronic rhinosinusitis with nasal polyps.

## Figures and Tables

**Table 1:** Research design: patient group selection criteria (ESS: endoscopic sinus surgery).

CRSwNP	Tissue IL-33 Study	CT Osteitis Severity Score Study
Number of patients	44	44
Medical institutions involved in research	(II) Department of Otorhinolaryngology, University Clinical Hospital of Railway Company, Cluj-Napoca, Romania Research Center for Functional Genomics, Biomedicine and Translational Medicine, Iuliu Hatieganu University of Medicine and Pharmacy, Cluj-Napoca, Romania	(II) Department of Otorhinolaryngology, University CF Cluj Clinical Hospital, Cluj-Napoca, Romania Department of Radiology, University CF Cluj Clinical Hospital, Cluj-Napoca, Romania Diagnostical and Interventional Radiology Department, Emergency Clinical Hospital, Bucharest, Romania
Inclusion criteria	Patients undergoing primary surgery for CRSwNP, diagnosed according to EPOS 2020 [11] criteria and with histological postoperative diagnosis	Patients undergoing primary ESS for CRSwNP, diagnosed according to EPOS 2020 [11] criteria, with histological postoperative diagnosis; Signed agreement on CT scan evaluation
Exclusion criteria	Patients under 18 years of age Pregnancy Revision surgery Anterior nasal trauma Sinonasal malignancies Systemic corticosteroids treatment in the previous month Macrolides treatment in the previous month Concurrent immunomodulatory therapy Insufficient sampling tissue	Mid and moderate disease CRSwNP Patients under 18 years of age Pregnancy Revision surgery Anterior nasal trauma Sinonasal malignancies Insufficient sampling tissue Disagreement on CT scan evaluation
Research analyses	Histopathology for CRSwNP confirmation diagnosis Assessing of tissue IL-33	Paranasal native CT scan Assessing of imaging osteitis severity scores

**Table 2:** The demographic characteristics and clinical parameters of the study cohort.

CRSwNP Cohort (*n* = 44)	Group Characteristics
Age (years)	47.47
Sex (male/female)	21/13
Asthma, *n* (%)	13 (29.54)
Hypereosinophilia, *n* (%)	18 (40.90)
Quality-of-life (SNOT-22 average value)	42.79
Allergies, *n* (%)	16 (36.36)
Endoscopy scores (average value)	4.36

**Table 3:** Global Osteitis Scoring Scale, after Georgalas et al. [5,18].

Scoring System	Evaluated Sites	Grading Description	Total Score
Global Osteitis Scoring Scale	10 sinuses (right and left frontal, anterior ethmoid, posterior ethmoid, maxillary, and sphenoid)	Grade 1: <50% sinus walls involvement and thickening < 3 mm wide	Composite score range 0–50
		Grade 2: <50% sinus walls involvement and thickening 3–5 mm wide	
		Grade 3: <50% of sinus walls involvement and thickening > 5 mm wide or >50% sinus walls involved and thickening < 3 mm wide	
		Grade 4: >50% of sinus walls involvement and thickening 3–5 mm wide	
		Grade 5: >50% of sinus walls involvement and thickening > 5 mm wide	

**Table 4:** Multiple linear regression model predicting CT osteitis score, with IL-33, asthma, and endoscopy sum score as independent variables.

Variable	Adjusted B	(95% CI)	*p*
IL-33 (pg/mL)	0.09	(0.05–0.12)	<0.001
Asthma	−0.67	(−1.51–0.16)	0.112
Endoscopy score	−0.16	(−0.49–0.17)	0.334

IL, interleukin; CI, confidence interval.

## Data Availability

No data available due to privacy concerns.
